# The efficacy and safety of cabazitaxel in the treatment of metastatic castration-resistant prostate cancer: a systematic review and network meta-analysis based on randomized controlled trials

**DOI:** 10.3389/fphar.2025.1586650

**Published:** 2025-07-17

**Authors:** Cong Shao, Quan Wan, Jia Guo, Zhuo Chen

**Affiliations:** ^1^Hebi Institute of Engineering and Technology, Henan Polytechnic University, Hebi, China; ^2^Department of Urology, Xi’an Electric Power Central Hospital, Xi’an, China; ^3^Department of Urology, The First Affiliated Hospital of Xi’an Jiao Tong University, Xi’an, China; ^4^Nursing Department, China Tibetology Research Center Beijing Hospital of Tibetan Medicine, Beijing, China

**Keywords:** prostate cancer, castration resistance, cabazitaxel, meta-analysis, systematic review

## Abstract

**Background:**

Cabazitaxel (CAB) has been approved for the treatment of patients with progressed metastatic castration-resistant prostate cancer (mCRPC) after receiving docetaxel. To assess the efficacy and safety of CAB in mCRPC patients through systematic review and network meta-analysis.

**Methods:**

Randomized controlled studies on the treatment of mCRPC with CAB in PUBMED, EMBASE, Cochrane, and Web of Science were searched. Relevant studies that met pre-set criteria were determined, and the quality of included studies was evaluated using the National Institutes of Health-Quality Assessment Tool. After the data was extracted, data analysis was conducted in R 4.3.2. Overall survival (OS), progression-free survival (PFS), and serious adverse events (SAEs) were used as the primary outcomes, and HR (hazard ratio) or RR (risk ratio) and their 95% confidence intervals (95% CrI) were calculated as effect sizes.

**Results:**

A total of 13 studies were included, involving 5,814 patients. The overall risk of bias for 13 studies was low. The results showed that CAB 25 mg/m^2^ significantly improved OS compared to androgen receptor pathway inhibitor (ARPI) (1.50 [1.30, 1.70]) and MIT (1.40 [1.20, 1.70]), but its efficacy was inferior to Lu-PSMA (0.42 [0.27, 0.67]) and therapeutic drug monitoring (TDM)-CAB (0.51 [0.38, 0.70]). CAB 25 mg/m^2^ could significantly improve PFS compared to CAB + CP (1.40 [1.10, 2.00]), ARPI (1.80 [1.50, 2.30], MIT (1.40 [1.20, 1.60]), but its efficacy was not as good as CAB 20 mg/m^2^ (0.92 [0.82, 1.00]), Lu-PSMA (0.58 [0.41, 0.80]), TDM-CAB (0.67 [0.51, 0.86]). In addition, compared to CAB 25 mg/m^2^, CAB + CP may significantly increase the risk of SAEs (3.10 [1.70, 5.90]).

**Conclusion:**

CAB is an effective treatment in mCRPC, and combining it with other treatment methods may enhance efficacy, but attention should be paid to the occurrence of adverse events.

## Introduction (background + purpose)

Prostate cancer (PCa) accounts for approximately 29% of male cancer cases and is the second leading cause of cancer-related deaths in men, second only to lung cancer (Siegel et al., 2024). Some studies predict that the number of people suffering from PCa will continue to increase in the future (Davies et al., 2019). For a long time, the treatment of PCa has been centered around targeting the androgen receptor (AR) (Siegel et al., 2024). However, when changes in the AR signaling pathway trigger drug resistance and drive the progression to metastatic castration-resistant prostate cancer (mCRPC), treating this disease is challenging (Author and Anonyms, 2021). Previous studies have shown that treatment options for mCRPC patients are limited with poor prognosis. Despite significant progress in treatment strategies recently, the 5-year relative survival rate for metastatic PCa remains hovering at only 34% (Dai et al., 2023). The prognosis of mCRPC patients is poor, with a median overall survival (OS) of approximately 3 years, making the treatment of this disease a major challenge (Ruiz de Porras et al., 2021). In 2004 (Bray et al., 2024), two Phase III clinical trials (Siegel et al., 2024) demonstrated that docetaxel can prolong the survival of patients with mCRPC. Subsequently, androgen receptor pathway inhibitors (ARPI) (abiraterone and enzalutamide), immunotherapy (siPuleucel-T), and bone-targeted radiopharmaceuticals (radium-223) were successively used to treat mCRPC (de Bono et al., 2011; Scher et al., 2012). However, only about 50% of PCa patients initially respond to docetaxel, and almost all patients develop drug resistance within 6–8 months (Lohiya et al., 2016). According to the 2025 ASCO Guidelines on systemic therapy in patients with mCRPC (Garje et al., 2025), PSMA-positive patients should receive either Lu-PSMA therapy or CAB. For PSMA-negative patients, CAB or radium 223 chemotherapy can be considered for the treatment of symptomatic bone metastases.

Cabazitaxel (CAB) is a paclitaxel with anti-tumor activity designed specifically for docetaxel-resistant models. A Phase III clinical trial (TROPIC, NCT00417079) showed that CAB significantly improved the OS of mCRPC (de Bono et al., 2010). A subsequent Phase III clinical trial (PROSELICA, NCT01308580) showed that CAB 20 mg/m2 was not inferior to CAB 25 mg/m^2^ and had better safety (Oudard et al., 2017).

At present, although the above studies have preliminarily assessed the efficacy and safety of CAB in the treatment of mCRPC, existing studies mainly focus on the comparison between different treatment regimens of CAB, as well as the differences between CAB treatment regimens and various non-CAB treatment regimens (such as DOC, (Lutetium-177 prostate-specific membrane antigen) Lu-PSMA and others). It is difficult to conduct a pairwise meta-analysis of the efficacy and safety of CAB in treating mCRPC, which makes it challenging to assess the role of CAB in mCRPC. This study aimed to systematically and comprehensively collect randomized controlled trials (RCTs) related to the treatment of mCRPC with CAB. Using a network meta-analysis method, we comprehensively compared direct and indirect evidence to evaluate the therapeutic effect of CAB in mCRPC.

## Materials and methods

### Registration

This study was conducted according to the Preferred Reporting Items for Systematic Reviews and Meta-Analyses (PRISMA) extension statement for network meta-analysis (NMA) and was pre-registered on the International Prospective Register of Systematic Reviews (PROSPERO) (Hutton et al., 2015) with the registration number of CRD42024493973.

### Literature retrieval

We searched Pubmed, Embase, Web of Science, and Cochrane Library databases from the date of database creation to May 2024, and made an update in June 2025. The main terms used to construct retrieval strategies included CAB, randomized controlled trials, and mCRPC. The complete search strategy is detailed in Supplementary Materials. No restrictions were imposed on language.

### Literature screening

All retrieved results were imported into the literature management software Endnote X9 for automatic and manual deduplication. The titles and abstracts of the remaining literature were quickly browsed to find studies that may meet the inclusion criteria. If it could not be fully confirmed by reading the title and abstract, a final decision may be made after the entire text was carefully read. Literature screening was independently conducted by two reviewers. The results of the two reviewers were cross-checked. If there were discrepancies, a third independent reviewer would be consulted to resolve them.

Inclusion criteria were proposed based on PICOS principles: 1) Study subjects: mCRPC patients or a population dominated by such patients (≥70%); 2) Intervention measures: treatment regimens involving CAB; 3) Control group: treatment regimens without CAB. 4) Outcome measures: one or more of the following relevant outcome measures shall be included: OS, progression-free survival (PFS), and incidence of grade 3–4 adverse events; 5) Study type: Randomized controlled trial (RCT).

Exclusion criteria: (1) Previous treatment with CAB; (2) Serious complications, severe psychological, family, social, or geographical conditions that may hinder the study protocol and subsequent plans; (3) When a randomized controlled trial was reported in multiple studies, those with short follow-up time, limited relevant information, and conference reports were excluded.

### Literature quality evaluation

According to the RCT section in the National Institutes of Health-Quality Assessment Tool (NIH-QAT, https://www.nhlbi.nih.gov/health-topics/study-quality-assessment-tools), the quality of the included studies was assessed. This tool included 14 questions to assess the randomization of literature to be evaluated, treatment allocation - two interrelated parts, blinding, similarity of groups at baseline, dropout, compliance, avoidance of other interventions, outcome measurement assessment, probability calculation, pre-specified outcomes, intention-to-treat analysis, and general guidance for determining the overall quality rating of controlled intervention studies. Finally, the quality of the included studies was evaluated as good, fair, or poor.

### Data extraction

After determining the final included studies, two independent researchers extracted the data based on a pre-designed sheet. Independent data extraction could minimize the risk of errors. When there were discrepancies in data extraction, they would be resolved by the third researcher. The following information was extracted from all included studies: (1) General information of articles: article title, publication year, author, country, language, and name of the randomized trial. (2) General information about patients: patient age, treatment measures for the included test and control groups, number of participants in both groups, patient activity status (Eastern Cooperative Oncology Group, ECOG), and baseline serum prostate-specific antigen (PSA) concentration. (3) Patient outcome measures.

### Statistical analysis

This study used R 4.3.2 to analyze the collected data, with the main application package of “gemtc” (1.0–1). The functions of “mtc.network”, “mtc.model”, “mtc.run”, “mtc.anohe”, “mtc.nodesplit” were used for the construction of network structures, model building, computation, heterogeneity analysis, and inconsistency testing. We assessed treatment efficacy and safety by calculating the hazard ratio (HR) or risk ratio (RR) of different interventions in different outcomes, as well as the corresponding 95% confidence intervals (CrIs). The results were presented in forest plots, league tables, and SUCRA values. The closer the surface under the cumulative ranking curve (SUCRA) value was to 1, the higher the ranking of the intervention measures.

## Results

### Literature screening

**FIGURE 1:**
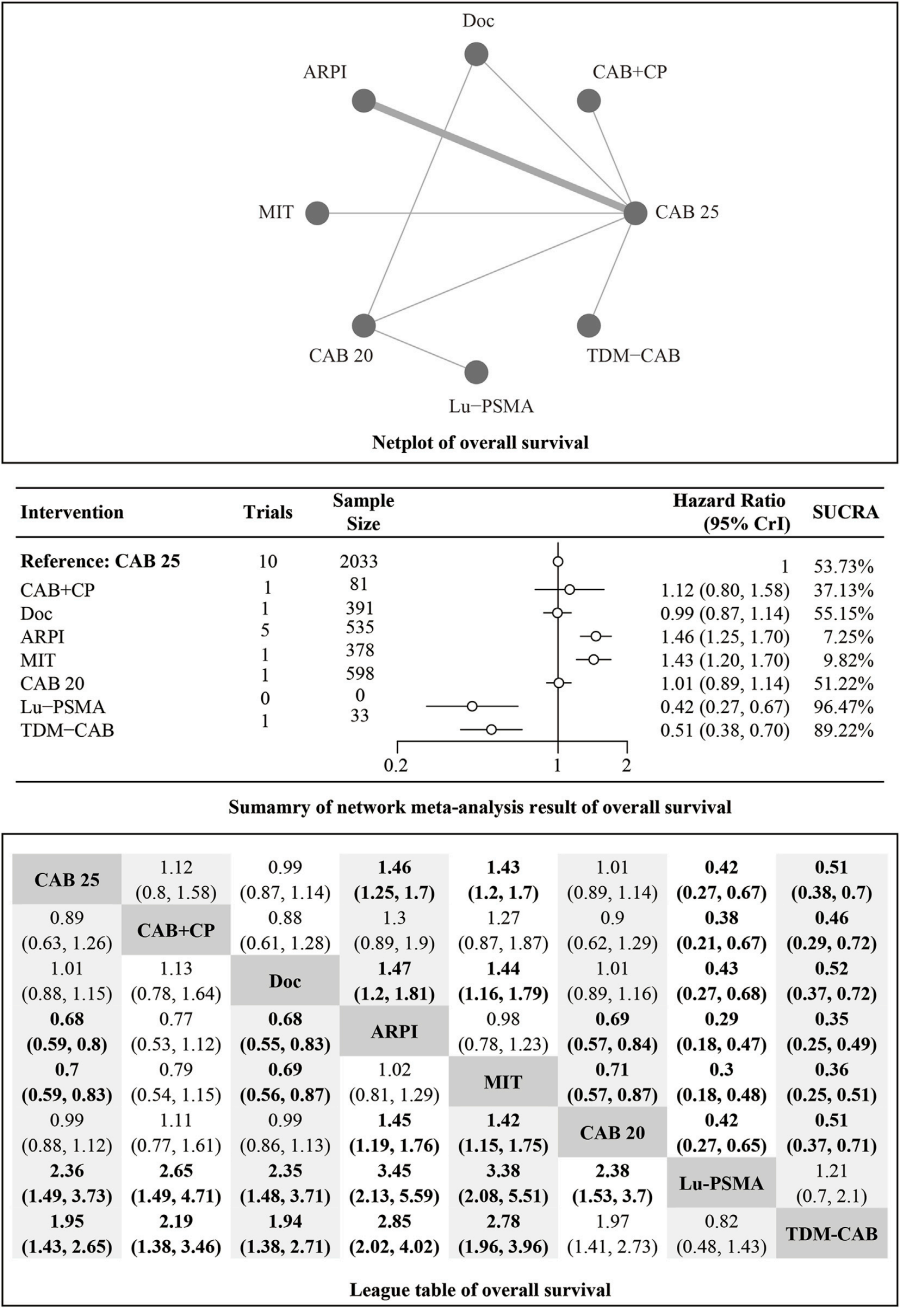
Mesh structure diagram of OS.

We retrieved 4,498 records, including 1,053 from PubMed, 2,611 from Embase, 227 from Cochrane, and 607 from Web of Science. All studies were imported into Endnote X9 and 125 duplicate articles were removed. Out of the remaining 4,373 articles, 4,296 were excluded based on established inclusion and exclusion criteria by quick viewing of the titles and abstracts of the articles. The full texts of the remaining 50 articles were downloaded and browsed carefully. 38 articles among them (including 13 articles of single-arm studies, 1 article lacking necessary outcome measures, 2 non-randomized controlled articles, 7 conference abstracts, 12 reviews, 1 duplicate publication, and 2 retrospective analyses) were removed. Finally, 12 articles were included (de Bono et al., 2010; Oudard et al., 2017; Corn et al., 2019; Suzuki et al., 2021; Fizazi et al., 2020; de Wit et al., 2019; Sternberg et al., 2021; Annala et al., 2021; Fleshner et al., 2023; Hofman et al., 2024; Eisenberger et al., 2017; Omlin et al., 2023). The process of literature screening is shown in Figure 1.

### Results of literature quality assessment

The overall quality of the included studies was good, and the main reason for the decreased quality was the lack of an effective description of blinding. In addition, some studies did not report whether the study protocol was pre-published, making it impossible to determine whether the study results or subgroups were designated in advance (Corn et al., 2019; Fizazi et al., 2020; Annala et al., 2021; Fleshner et al., 2023; Omlin et al., 2023). The detailed evaluation results are shown in Supplementary Materials.

### Basic information of included studies

As of June 2024, this network meta-analysis included a total of 12 studies, all of which were published between 2010 and 2024. The meta-analysis involved a total of 5,814 patients, with 3,832 patients assigned to the CAB group and 1,982 patients assigned to the other treatment group. ARPI was used in six trials (Suzuki et al., 2021; Fizazi et al., 2020; de Wit et al., 2019; Sternberg et al., 2021; Annala et al., 2021; Fleshner et al., 2023), while the treatment measures used in other trials were mitoxantrone (de Bono et al., 2010), docetaxel (Oudard et al., 2017), CAB and carboplatin (Buonerba et al., 2014), therapeutic drug monitoring (TDM)-CAB (Omlin et al., 2023), and Lu-PSMA (Hofman et al., 2024). Among them, in the Eisenberger (Eisenberger et al., 2017) and Oudard (Oudard et al., 2017) trials, the dose of CAB was 20 mg/m^2^ every 3 weeks, while in other trials, the dose of CAB was 25 mg/m^2^ every 3 weeks. Before the start of the trials, the majority of patients had serum PSA concentrations at medium to low levels. Patients with ECOG status of 0 or 1 accounted for over 90% of the total number of patients, as shown in Table 1.

**TABLE 1:** Demographic characteristics of included trials.

Study	NCT registration	Region	Intervention	Sample size	ECOG	NIH QAT
Corn 2019	NCT01505868	United States	CAB (25 mg/m^2^) + CP	81	0/1–2:22/59	Yes
			CAB (20–25 mg/m^2^)	79	0/1–2:21/58	
Suzuki 2021	NCT02485691	Japanese	ARPI	107	0/1–104	Yes
			CAB (25 mg/m^2^) Q3W	115	0/1–111	
de Bono 2010	NCT00417079	United States	MIT	378	0/1–344	Yes
			CAB (25 mg/m^2^) Q3W	377	0/1–350	
Fizazi 2020	NCT02485691	France	CAB (25 mg/m^2^) Q3W	129	0/1–2:123/6	Yes
			ARPI	126	0/1–2:119/7	
Wit 2019	NCT02485691	United States	CAB (25 mg/m^2^) Q3W	129	0/1–2:123/6	Yes
			ARPI	126	0/1–2:119/7	
Sternberg 2021	NCT02485691	United States	CAB (25 mg/m^2^) Q3W	129	0/1–2:125/4	Yes
			ARPI	126	0/1–2:122/4	
Annala 2021	NCT02254785	Canada	CAB (25 mg/m^2^) Q3W	45	0/1–41	Yes
			ARPI	50	0/1–48	
Fleshner 2022	NCT02543255	American	CAB (25 mg/m^2^) Q3W	37		Yes
			ARPI	33		
Hofman 2024	NCT03392428	Australia	CAB (20 mg/m^2^) Q3W	101	0/1/2–42:53/4	Yes
			Lu-PSMA	99	0/1/2–44:52/4	
Eisenberger 2017	NCT01308580	American	CAB (20 mg/m^2^) Q3W	598	0/1/2–539/59	Yes
			CAB (25 mg/m^2^) Q3W	602	0/1/2–540/62	
Omlin 2023	NCT02561948	American	CAB (25 mg/m^2^) Q3W	40	0/1/2–18/20/2	Yes
			TDM-CAB	33	0/1/2–16/16/1	
Oudard 2017	NCT01308567	American	CAB (20 mg/m^2^) Q3W	389	0/1-2–370/19	Yes
			CAB (25 mg/m^2^) Q3W	388	0/1-2–376/12	
			DOC(75 mg/m^2^) Q3W	391	0/1-2–374/17	

Abbreviation: CABQ3W, cabazitaxel every 3 weeks; CP, carboplation; MIT, mitoxantrone; ARPI, androgen receptor pathway inhibitors; Lu-PSMA, Lutetium-177 [177Lu]Lu-PSMA-617, prostate-specific membrane antigen; DOC, docetaxel; TDM-CAB, therapeutic drug monitoring-cabazitaxel; ECOG, Eastern Cooperative Oncology Group.

### Statistical analysis results

#### OS

**FIGURE 2:**
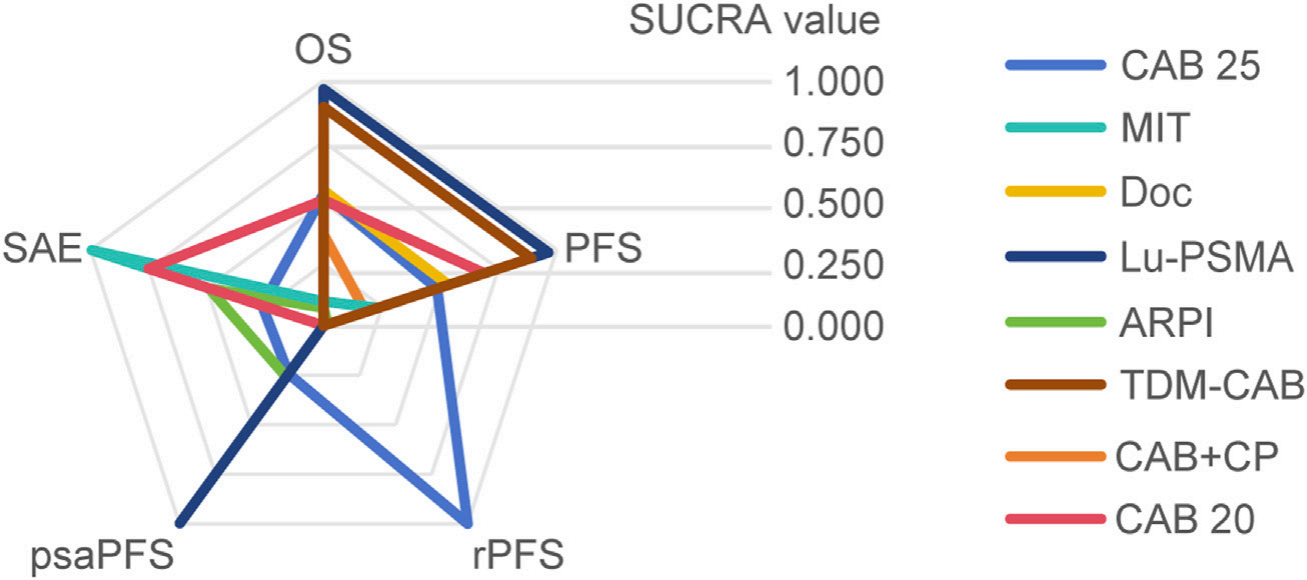
Summary of SUCRA value.

Regarding the OS, there were 11 studies with a total of 4,049 patients included in the analysis. A total of 8 intervention measures were adopted. The network structure diagram i shown in Figure 1. The inconsistency test of heterogeneity analysis and node split method indicated that the network meta-analysis met the homogeneity hypothesis and consistency hypothesis (as shown in Supplementary Figure S1). The results presented in the article are based on a fixed effects model. To assess whether the pooling method influenced the stability of the findings, an additional analysis was performed using a fixed effects model, which showed no significant differences (as shown in Supplementary Figure S1). Compared with CAB 25 mg/m^2^, Lu-PSMA (HR [95% CrI] = 0.42 [0.27, 0.67]), TDM-CAB (HR [95% CrI] = 0.51 [0.38, 0.70]) can significantly improve OS. No differences in OS with DOC (HR [95% CrI] = 0.99 [0.87, 1.10]), CAB 20 mg/m^2^ (HR [95% CrI] = 1.00 [0.89, 1.10]), and CAB + CP (HR [95% CrI] = 1.10 [0.80, 1.60]). ARPI (HR [95% CrI] = 1.50 [1.30, 1.70]), MIT (HR [95% CrI] = 1.40 [1.20, 1.70]) were inferior to CAB 25 mg/m^2^. The forest plot of OS is illustrated in Figure 1. The league table and SUCRA values showed a similar trend to the forest plot. No differences were noted between CAB 20 mg/m^2^, CAB 25 mg/m^2^, and DOC, and all were more effective than ARPI and MIT, and were inferior to Lu-PSMA and TDM-CAB. The league table was detailed in Table 2. OS. The probability ranking of SUCRA values was as follows: Lu-PSMA (96.47%), TDM-CAB (89.23%), DOC (55.15%), CAB 20 mg/m^2^ (51.22%), CAB + CP (37.13%), MIT (9.82%), ARPI (7.25%), and CAB 25 mg/m^2^ of control group (53.73%) (Figure 2).

**TABLE 2:** League table of overall survival.

CAB25	1.12 (0.8, 1.58)	0.99 (0.87, 1.14)	1.46 (1.25, 1.7)	1.43 (1.2, 1.70)	1.01 (0.89, 1.14)	0.42 (0.27, 0.67)	0.51 (0.38, 0.70)
0.89 (0.63, 1.26)	CAB + CP	0.88 (0.61, 1.28)	1.30 (0.89, 1.9)	1.27 (0.87, 1.87)	0.90 (0.62, 1.29)	0.38 (0.21,0.67)	0.46 (0.29,0.72)
1.01 (0.88, 1.15)	1.13 (0.78, 1.64)	Doc	1.47 (1.2,1.81)	1.44 (1.16,1.79)	1.01 (0.89, 1.16)	0.43 (0.27,0.68)	0.52 (0.37,0.72)
0.68 (0.59,0.80)	0.77 (0.53, 1.12)	0.68 (0.55,0.83)	ARPI	0.98 (0.78, 1.23)	0.69 (0.57,0.84)	0.29 (0.18,0.47)	0.35 (0.25,0.49)
0.70 (0.59,0.83)	0.79 (0.54, 1.15)	0.69 (0.56,0.87)	1.02 (0.81, 1.29)	MIT	0.71 (0.57,0.87)	0.30 (0.18,0.48)	0.36 (0.25,0.51)
0.99 (0.88, 1.12)	1.11 (0.77, 1.61)	0.99 (0.86, 1.13)	1.45 (1.19,1.76)	1.42 (1.15,1.75)	CAB20	0.42 (0.27,0.65)	0.51 (0.37,0.71)
2.36 (1.49,3.73)	2.65 (1.49,4.71)	2.35 (1.48,3.71)	3.45 (2.13,5.59)	3.38 (2.08,5.51)	2.38 (1.53,3.70)	Lu-PSMA	1.21 (0.70, 2.10)
1.95 (1.43,2.65)	2.19 (1.38,3.46)	1.94 (1.38,2.71)	2.85 (2.02,4.02)	2.78 (1.96,3.96)	1.97 (1.41,2.73)	0.82 (0.48, 1.43)	TDM-CAB

### Median PFS

**FIGURE 3:**
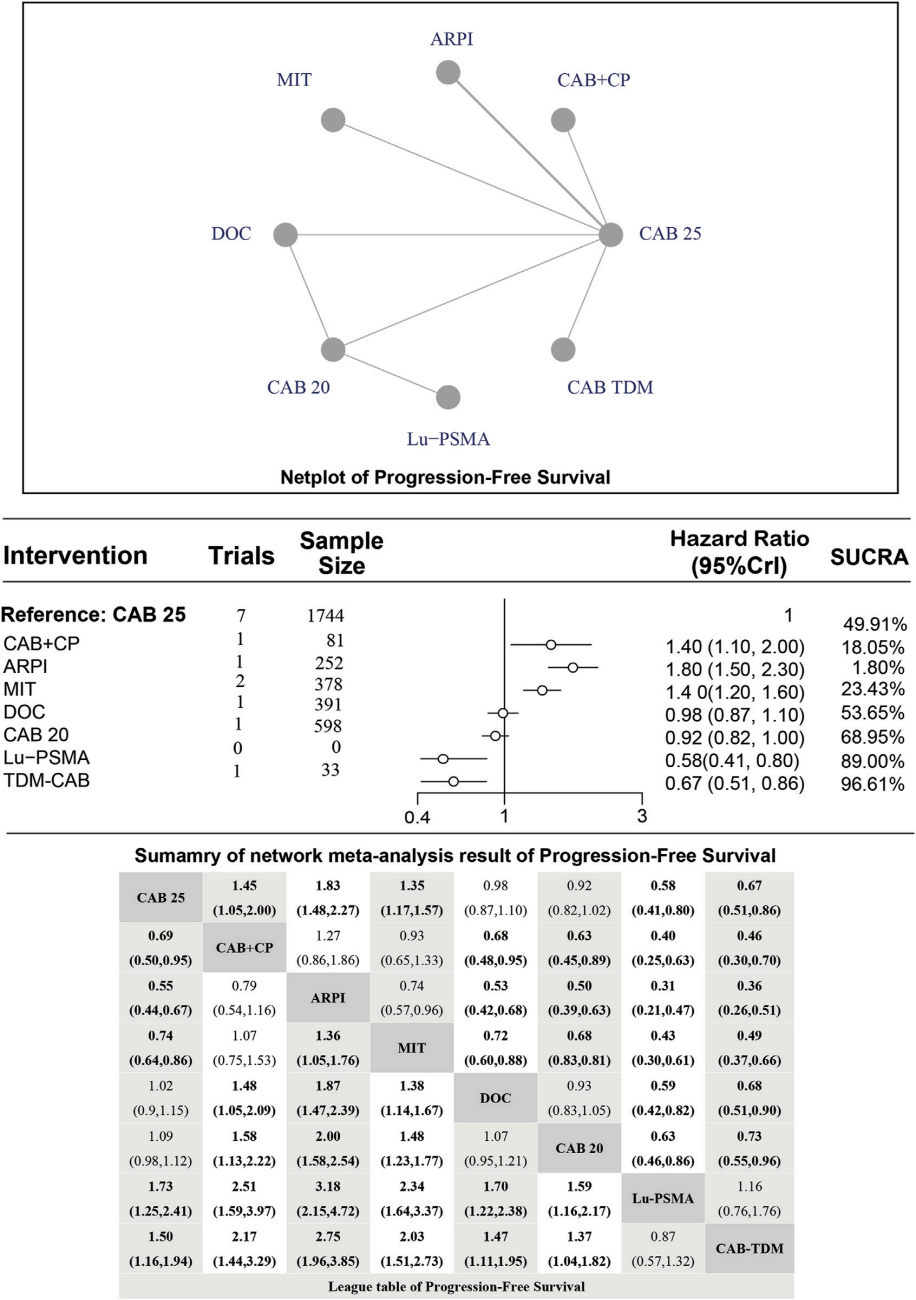
Mesh structure diagram of PFS.

Regarding the PFS, there were 8 studies with a total of 3,477 patients included in the analysis. A total of 7 intervention measures were adopted. The network structure diagram of PFS is shown in Figure 3. The inconsistency test of heterogeneity analysis and node split method indicated that the network meta-analysis met the homogeneity hypothesis and consistency hypothesis (as shown in Supplementary Figure S2). The results presented in the article are based on a fixed effects model. To assess whether the pooling method influenced the stability of the findings, an additional analysis was performed using a fixed effects model, which showed no significant differences (as shown in Supplementary Figure S2). Compared with CAB 25 mg/m^2^, CAB 20 mg/m^2^ (HR [95% CrI] = 0.92 [0.82, 1.00]), Lu-PSMA (HR [95% CrI] = 0.58 [0.41, 0.80]), and TDM-CAB (HR [95% CrI] = 0.67 [051, 0.86]) can significantly improve PFS; There were no differences in PFS with DOC (HR [95% CrI] = 0.98 [0.87, 1.10]). CAB + CP (HR [95% CrI] = 1.40 [1.10, 2.00]), ARPI (HR [95% CrI] = 1.80 [1.50, 2.30], and MIT (HR [95% CrI] = 1.40 [1.20, 1.60]) were inferior to CAB 25 mg/m^2^. The forest plot of PFS is depicted in Figure 3. The league table and SUCRA values showed a similar trend to the forest plot. CAB 25 mg/m^2^ was more efficacious than CAB + CP, ARPI and MIT, and inferior to DOC, CAB 20 mg/m^2^, Lu-PSMA and TDM-CAB. The probability ranking of SUCRA values was as follows: TDM-CAB (96.41%), Lu-PSMA (89.00%), CAB 20 mg/m^2^ (68.95%), DOC (53.65%), MIT (23.43%), CAB + CP (18.05%), ARPI (1.80%) and CAB 25 mg/m^2^ of control group (48.72%). The league table of PFS is detailed in Table 3 and Figure 2.

**TABLE 3:** League table of progression-free survival.

CAB 25	1.45 (1.05, 2.00)	1.83 (1.48, 2.27)	1.35 (1.17, 1.57)	0.98 (0.87, 1.10)	0.92 (0.82, 1.02)	0.58 (0.41, 0.80)	0.67 (0.51, 0.86)
0.69 (0.50,0.95)	CAB + CP	1.27 (0.86, 1.86)	0.93 (0.65, 1.33)	0.68 (0.48,0.95)	0.63 (0.45,0.89)	0.40 (0.25,0.63)	0.46 (0.30,0.70)
0.55 (0.44,0.67)	0.79 (0.54, 1.16)	ARPI	0.74 (0.57, 0.96)	0.53 (0.42,0.68)	0.50 (0.39,0.63)	0.31 (0.21,0.47)	0.36 (0.26,0.51)
0.74 (0.64,0.86)	1.07 (0.75, 1.53)	1.36 (1.05,1.76)	MIT	0.72 (0.60,0.88)	0.68 (0.83,0.81)	0.43 (0.30,0.61)	0.49 (0.37,0.66)
1.02 (0.9, 1.15)	1.48 (1.05,2.09)	1.87 (1.47,2.39)	1.38 (1.14,1.67)	DOC	0.93 (0.83, 1.05)	0.59 (0.42,0.82)	0.68 (0.51,0.90)
1.09 (0.98, 1.12)	1.58 (1.13,2.22)	2.00 (1.58,2.54)	1.48 (1.23,1.77)	1.07 (0.95, 1.21)	CAB 20	0.63 (0.46,0.86)	0.73 (0.55,0.96)
1.73 (1.25,2.41)	2.51 (1.59,3.97)	3.18 (2.15,4.72)	2.34 (1.64,3.37)	1.70 (1.22,2.38)	1.59 (1.16,2.17)	Lu-PSMA	1.16 (0.76, 1.76)
1.50 (1.16,1.94)	2.17 (1.44,3.29)	2.75 (1.96,3.85)	2.03 (1.51,2.73)	1.47 (1.11,1.95)	1.37 (1.04,1.82)	0.87 (0.57, 1.32)	CAB-TDM

### Median radiographic PFS (rPFS)

**FIGURE 4:**
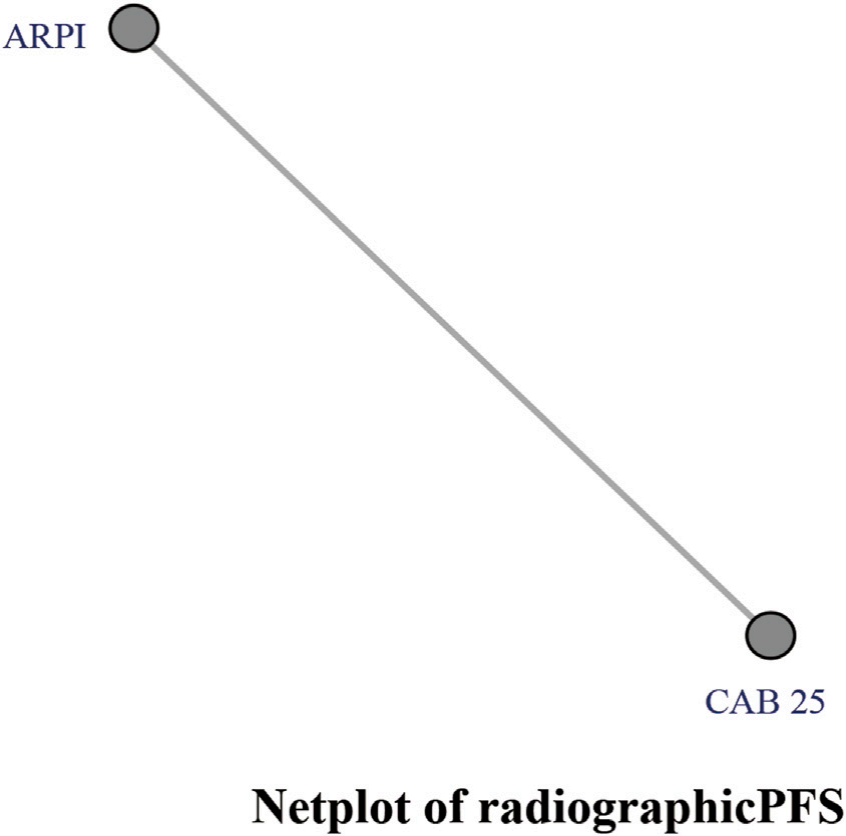
Mesh Diagram of rPFS.

Regarding the rPFS, there were 2 studies with a total of 477 patients included in the analysis. A total of 2 intervention measures were adopted. The network structure diagram of rPFS is illustrated in Figure 4. The inconsistency test of heterogeneity analysis and node split method indicated that the network meta-analysis did not form a closed network loop, and therefore no analysis was conducted.

### Median psaPFS

**FIGURE 5:**
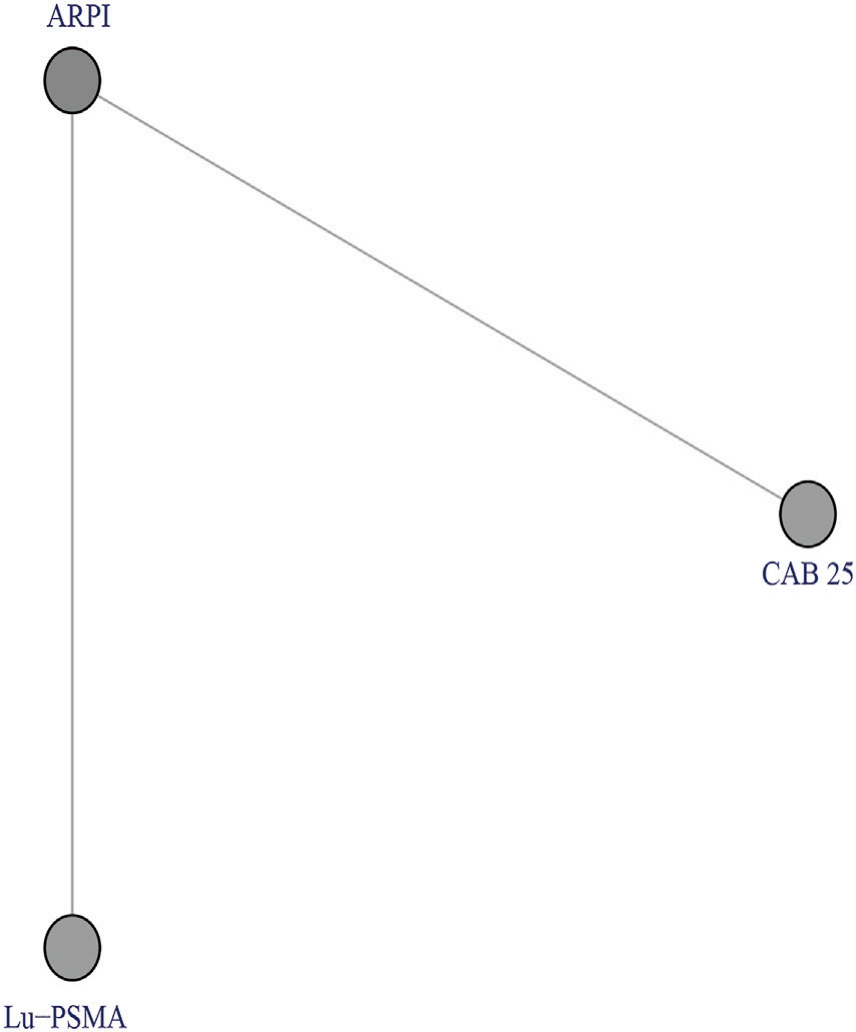
Mesh Diagram of psaPFS.

Regarding the psaPFS, there were 2 studies with a total of 313 patients included in the analysis. A total of 3 intervention measures were adopted. The network structure diagram of psaPFS is depicted in Figure 5. The inconsistency test of heterogeneity analysis and node split method indicated that the network meta-analysis did not form a closed network loop, and therefore no analysis was conducted.

### Incidence of serious adverse events (SAEs)

**FIGURE 6:**
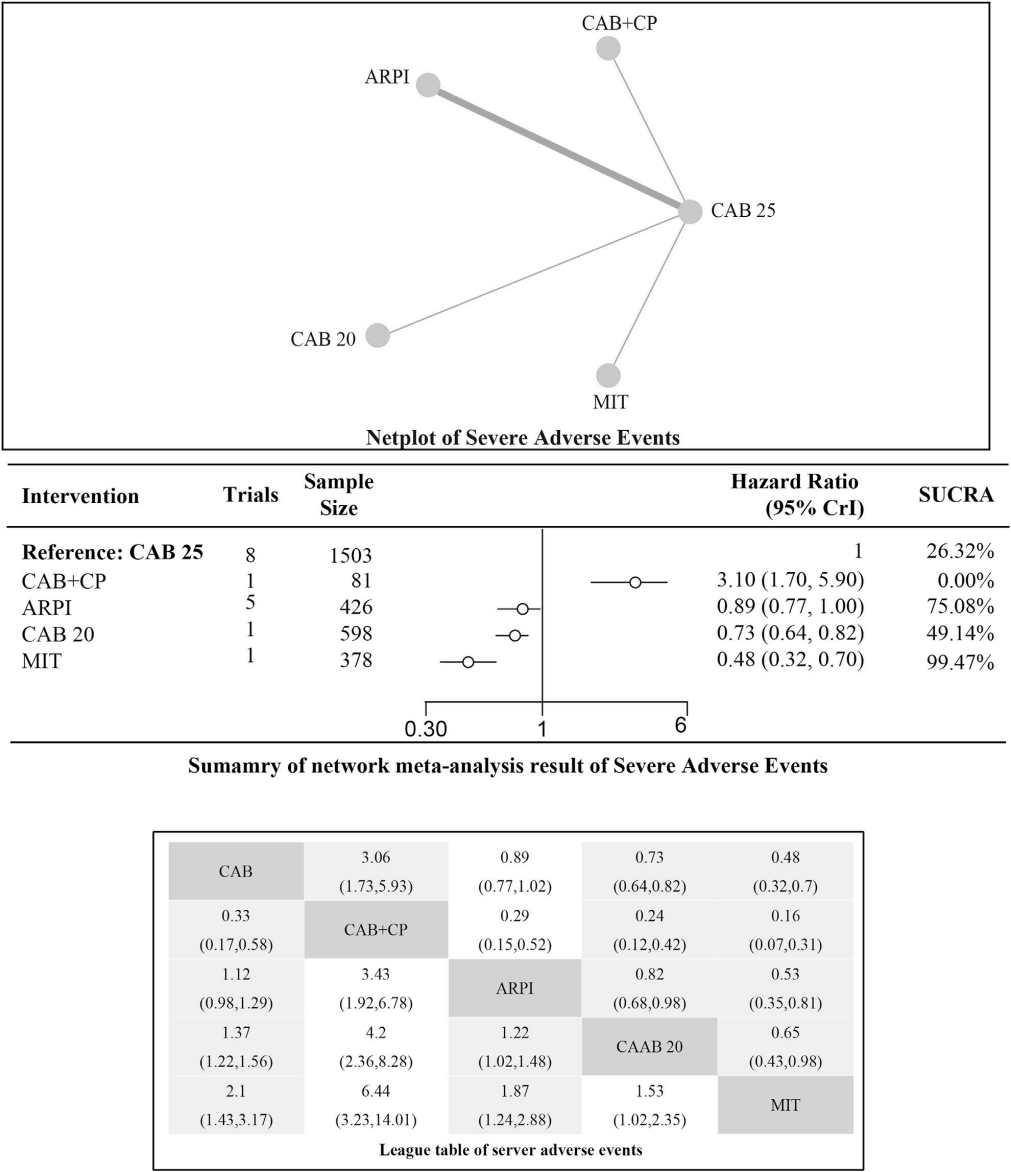
Mesh diagram of SAEs.

Regarding the incidence of SAEs (Grade 3–4 adverse events), there were 8 studies with a total of 3,012 patients included in the analysis. A total of 4 intervention measures were adopted. The network structure diagram of SAE is illustrated in Figure 6. The inconsistency test of heterogeneity analysis and node split method indicated that the network meta-analysis met the homogeneity hypothesis and consistency hypothesis (as shown in Supplementary Figure S3). The results presented in the article are based on a fixed effects model. To assess whether the pooling method influenced the stability of the findings, an additional analysis was performed using a fixed effects model, which showed no significant differences (as shown in Supplementary Figure S3) (I^2^<50%, P > 0.05). Compared with CAB 25 mg/m^2^, ARPI (RR [95% CrI] = 0.89 [0.77, 1.00]), MIT (RR [95% CrI] = 0.48 [0.32, 0.70]), and CAB 20 mg/m^2^ (RR [95% CrI] = 0.73 [0.64, 0.82]) can significantly reduce SAEs. CAB + CP (RR [95% CrI] = 3.10 [1.70, 5.90]) was inferior to CAB 25 mg/m^2^. The forest plot of SAEs is shown in Figure 6. The league table and SUCRA values showed a similar trend to the forest plot. CAB 25 mg/m^2^ was superior to CAB + CP and CAB 20 mg/m^2^, but inferior to ARPI and MIT. The probability ranking of SUCRA values was as follows: MIT (99.47%), ARPI (75.08%), CAB 20 mg/m^2^ (49.14%), CAB + CP (0.0%), and CAB 25 mg/m^2^ of control group (26.32%). The league table of SAEs is detailed in Table 4 and Figure 2.

**TABLE 4:** League table of server adverse events.

CAB 25	3.06 (1.73, 5.93)	0.89 (0.77, 1.02)	0.73 (0.64, 0.82)	0.48 (0.32, 0.70)
0.33 (0.17, 0.58)	CAB + CP	0.29 (0.15, 0.52)	0.24 (0.12, 0.42)	0.16 (0.07, 0.31)
1.12 (0.98, 1.29)	3.43 (1.92, 6.78)	ARPI	0.82 (0.68, 0.98)	0.53 (0.35, 0.81)
1.37 (1.22, 1.56)	4.20 (2.36, 8.28)	1.22 (1.02, 1.48)	CAB 20	0.65 (0.43, 0.98)
2.10 (1.43, , 3.17)	6.44 (3.23, 14.01)	1.87 (1.24, 2.88)	1.53 (1.02, 2.35)	MIT

## Discussion

This study found that CAB regimens, except for TDM-CAB, had no significant difference from DOC, and were superior to ARPI and MIT, but inferior to Lu-PSMA. The combination of CP and CAB may reduce PFS, while the combination of TDM method and CAB regimen could significantly improve patient prognosis. There was no significant difference among all the intervention measures mentioned above in the occurrence of SAEs.

### CAB vs. DOC

This study found that CAB and DOC have similar effects on OS and PFS in mCRPC patients, but CAB may be safer. Although theoretically, CAB may be more effective than DOC, the findings of this study are not curious based on conflicting previous study results. CAB is a new compound obtained by modifying the structure of docetaxel (Shi et al., 2020). Its structural changes reduce the inhibitory effect on P-glycoprotein (P-gp), enhance its cell membrane penetration ability, and compromise efflux tendency, thereby improving its killing efficiency against tumor cells (Sun et al., 2023). In previous studies, CAB was found to be less resistant to DOC and could serve as a rescue treatment for DOC-resistant patients (Robin et al., 2024; Beer et al., 2017). Although the therapeutic effect of CAB in mCRPC is highly anticipated, there is still controversy over which is better or worse between CAB and DOC. It has been demonstrated that CAB does not improve OS compared to DOC (Richter et al., 2017). The FIRSTANA study (de Bono et al., 2010) found that in newly treated mCRPC patients undergoing chemotherapy, CAB 20 mg/m^2^ or CAB 25 mg/m^2^ can significantly improve OS compared to DOC75, but there is no significant difference in PFS. In Kujime Y et al.‘s study, it was found that compared to DOC, CAB can significantly reduce side effects such as nausea, diarrhea, and neutropenia, and this result has also been confirmed in Richter I rt al.‘s study (Richter et al., 2017; Kujime et al., 2024). Based on the results of this study and previous evidence, given their similar therapeutic efficacy and the safer profile of CAB, CAB may be preferred over DOC in mCRPC. However, the relative advantages and disadvantages between the two still remain to be confirmed in future research.

### CAB 20 mg/m^2^ vs. CAB 25 mg/m^2^

CAB is an effective drug for the treatment of mCRPC, with a standard dose of 25 mg/m^2^ every 3 weeks. However, considering the serious side effects possibly induced by high doses, recent studies have explored the efficacy and safety of a lower dose of 20 mg/m^2^, but relevant studies have not confirmed whether CAB 20 mg/m^2^ is not inferior to CAB 25 mg/m^2^. A study showed that (Urabe et al., 2023) the PFS and OS of the CAB 20 mg/m^2^ group were significantly shorter than those of the CAB 25 mg/m^2^ group, while the difference in the incidence of adverse events was not statistically significant. Another study shows that (Baciarello et al., 2022) CAB 25 mg/m^2^ has significant clinical efficacy in the treatment of mCRPC; CAB 20 mg/m^2^ may achieve good therapeutic effects in some patients, but its overall efficacy may be slightly inferior to CAB 25 mg/m^2^, although CAB 20 mg/m^2^ shows better tolerability (Heidenreich and Pfister, 2018). However, another study found that patients in the 20 mg/m^2^ dose group had a significantly lower risk of severe hematological toxicity compared to those in the 25 mg/m^2^ dose group (Urabe et al., 2023). These studies indicated that dose reduction had a significant impact on the therapeutic efficacy of CAB, but the safety was still questionable. Based on current evidence and the results of this study, in clinical practice, a dose of 20 mg/m^2^ may be a safer choice for patients with poor physical condition and high risk of hematological toxicity, but a dose of 25 mg/m^2^ may be more appropriate for other patients as it can provide better prognosis (Oudard et al., 2017). In future study directions, although current studies have provided valuable information about different doses of CAB, further research is still needed to clarify the optimal dose. Future clinical trials can also focus on the efficacy and safety of low-dose CAB in specific patient populations, such as those at high risk of blood toxicity reactions. Low-dose CAB combination therapy strategies shall be simultaneously considered to improve efficacy and reduce side effects.

### Lu-PSMA vs. CAB

The VISION (Fizazi et al., 2023) and CARD trials, respectively, showed that both Lu-PSMA and CAB were superior to the use of second-line ARPIs or optimal supportive therapy. However, Lu-PSMA may have more advantages in improving patient prognosis. Lu-PSMA (Sartor et al., 2021) (Lutetium-177 labeled prostate-specific membrane antigen ligand) is a radionuclide therapy that achieves precise targeting of tumors by targeting PSMA receptors over-expressed on the surface of PCa cells. This therapy not only efficiently kills cancer cells, but also maximizes the protection of normal tissues and reduces the occurrence of side effects. Multiple studies have shown that (Fleshner et al., 2023; Sartor et al., 2021; Hennrich and Eder, 2022; Wenzel et al., 2025; Gillessen et al., 2025), Lu-PSMA therapy has significant therapeutic effects on advanced or metastatic PCa, and can significantly prolong the patient’s PFS and OS. Studies have shown that (Hofman et al., 2024; Hofman et al., 2019) Lu-PSMA therapy can significantly reduce PSA levels, indicating its effective control of tumor burden. In Fizaz et al.‘s (Fizazi et al., 2023) study, Lu-PSMA combined with standard care improved patients’ quality of life and reduced the time for bone events. Common side effects induced by Lu-PSMA therapy include fatigue, dry mouth, nausea, anemia, decreased appetite, and constipation (Fallah et al., 2023). The most common laboratory abnormalities in ≥30% of patients receiving Lu-PSMA treatment are lymphopenia, hemoglobin reduction, leukopenia, thrombocytopenia, calcium reduction, and sodium reduction, most of which are mild to moderate and can be effectively controlled through appropriate management. A systematic review suggests that (Sartor et al., 2021) Lu-PSMA therapy is safe with a low incidence of SAEs, prolongs the time to worsening of health-related quality of life and pain, and delays biochemical progression. Specifically (Hofman et al., 2024), the most common grade 3–4 adverse events in the Lu-PSMA and CAB groups were neutropenia (33% vs. 53%), thrombocytopenia, anemia, diarrhea, and fatigue. Compared with CAB, Lu-PSMA was associated with lower rates of rash, palmar-plantar erythrodysesthesia, dyspepsia, dizziness, urinary symptoms, and diarrhea. Regarding treatment accessibility, only one study in the existing literature has investigated the use of Lu-PSMA in mCRPC. Compared to other established therapies, key aspects of its clinical application—including optimal dosing, timing of administration, and treatment duration—remain undefined in mCRPC patients. Further research is needed to establish standardized treatment protocols. These results support the use of Lu-PSMA. In addition, this study found that Lu-PSM and TDM-CAB have similar efficacy and safety profiles. Currently, there is a lack of direct comparative evidence between the two treatment options, and future clinical studies can further explore this aspect. Furthermore, previous studies have explored the combination of radiotherapy and chemotherapy regimens for mCRPC patients. Thus, future studies are desired to combine these two regimens.

### TDM-CAB

This study found that TDM-CAB can significantly improve the prognosis of patients compared to conventional CAB use. There is a relationship between an increase in area under the curve (AUC) of concentration-time of CAB and a decrease in neutrophil percentage (Diéras et al., 2013). Therefore, a uniform dose of CAB based on body surface area (BSA) cannot explain the specific pharmacology of the drug. TDM refers to the method of regularly measuring drug concentrations in patients’ bodies to ensure that drug doses are both safe and effective. By monitoring the levels of CAB in plasma, doctors can adjust the dosage according to the specific situation of each patient to ensure optimal treatment outcomes while minimizing toxic reactions. At present, there is relatively limited research on the TDM of CAB, but existing studies have shown that TDM may improve the prognosis of patients. It has been reported that (Omlin et al., 2023) TDM regimens can provide better survival benefits and reduce the risk of adverse reactions for patient populations carrying specific gene mutations. In addition, another study reveals that (Eisenberger et al., 2017) dose adjustments guided by TDM can improve patients’ tolerance to CAB, thereby prolonging PFS.

The optimal administration strategy (weekly vs. every 3 weeks) or optimal average dose (CAB, albumin bound paclitaxel) for taxane drugs has not been fully resolved (Muth et al., 2021). Previous studies on dose range were mainly limited to Phase I clinical trials of small sample sizes, and could not effectively assess the interaction between dose differences and population differences (Papachristos et al., 2023). Although TDM provides a new direction for optimizing treatment with CAB, its practical application is still challenging. On the one hand, the implementation of TDM requires additional laboratory testing and professional support, which may increase treatment costs. Therefore, it is necessary to assess its long-term cost-effectiveness. On the other hand, there may be differences in measurement methods and standards between different laboratories, which may affect the consistency and reliability of TDM results. In Omlin’s (Omlin et al., 2023) study, the pharmacokinetic target for cabazitaxel was established based on the concentration-time area under the curve (AUC). Maintaining an AUC between 0.8 and 1.2 mgh/L was associated with reduced hematologic toxicity and consequently improved patient prognosis. However, as this target is currently derived from a single study, further verification may be required in future investigations. Establishing unified standards and guidelines based on more clinical research is crucial for promoting TDM.

### Strengths and limitations

#### Strengths

First, this is the first meta-analysis assessing the efficacy and safety of CAB in mCRPC, providing important guidance for clinical decision-making. Second, the included articles are all randomized controlled trials published in high-scoring journals, with high credibility of the conclusions.

#### Limitations

First, the number of included literature in this study is limited, and further verification of the literature may be needed in the future. Second, some outcome measures are not analyzed due to no closed loop, making it impossible to conduct inconsistency tests, and the resulting biases need to be further tested. Third, the lack of detailed blinding procedures represents a significant methodological limitation in the included RCTs, which may compromise the reliability of our findings. Future studies should strictly adhere to the CONSORT (Consolidated Standards of Reporting Trials) Statement for comprehensive reporting of blinding design. Fourth, none of the included studies conducted subgroup analyses to examine the potential influence of patient characteristics (such as PSA levels, ECOG performance status, BMI (Sforza et al., 2020), or other baseline prognostic factors in mCRPC patients) or comorbidities on treatment outcomes. Given the limited number of available studies, our analysis could not adequately address these issues. Future research should incorporate prespecified subgroup analyses or prospective registry studies to further investigate these factors. Fifth, while hematologic toxicity and other adverse events are commonly reported in current studies of CAB or Lu-PSMA treatment for mCRPC patients, the identification of high-risk populations remains unclear. Subsequent studies should focus on identifying relevant risk factors to optimize prevention and management strategies for treatment-related adverse events, including hematologic toxicities.

## Conclusion

The results of this study indicate that CAB is an effective and relatively safe treatment in mCRPC, and TDM can maximize its therapeutic effects and safety. However, its relationship with docetaxel, other combination therapies, and different doses still needs to be further confirmed. Given the limited number of articles included in this study, the results need to be interpreted with caution, and further research is still necessary in the future.

## Data Availability

The original contributions presented in the study are included in the article/Supplementary Material, further inquiries can be directed to the corresponding author.
