# Supplementary figures and images for: The efficacy and safety of cabazitaxel in the treatment of metastatic castration-resistant prostate cancer: a systematic review and network meta-analysis based on randomized controlled trials
Source: Front Pharmacol. 2025 Jul 17;16:1586650. doi: 10.3389/fphar.2025.1586650 (PMC12310731; doi:10.3389/fphar.2025.1586650)

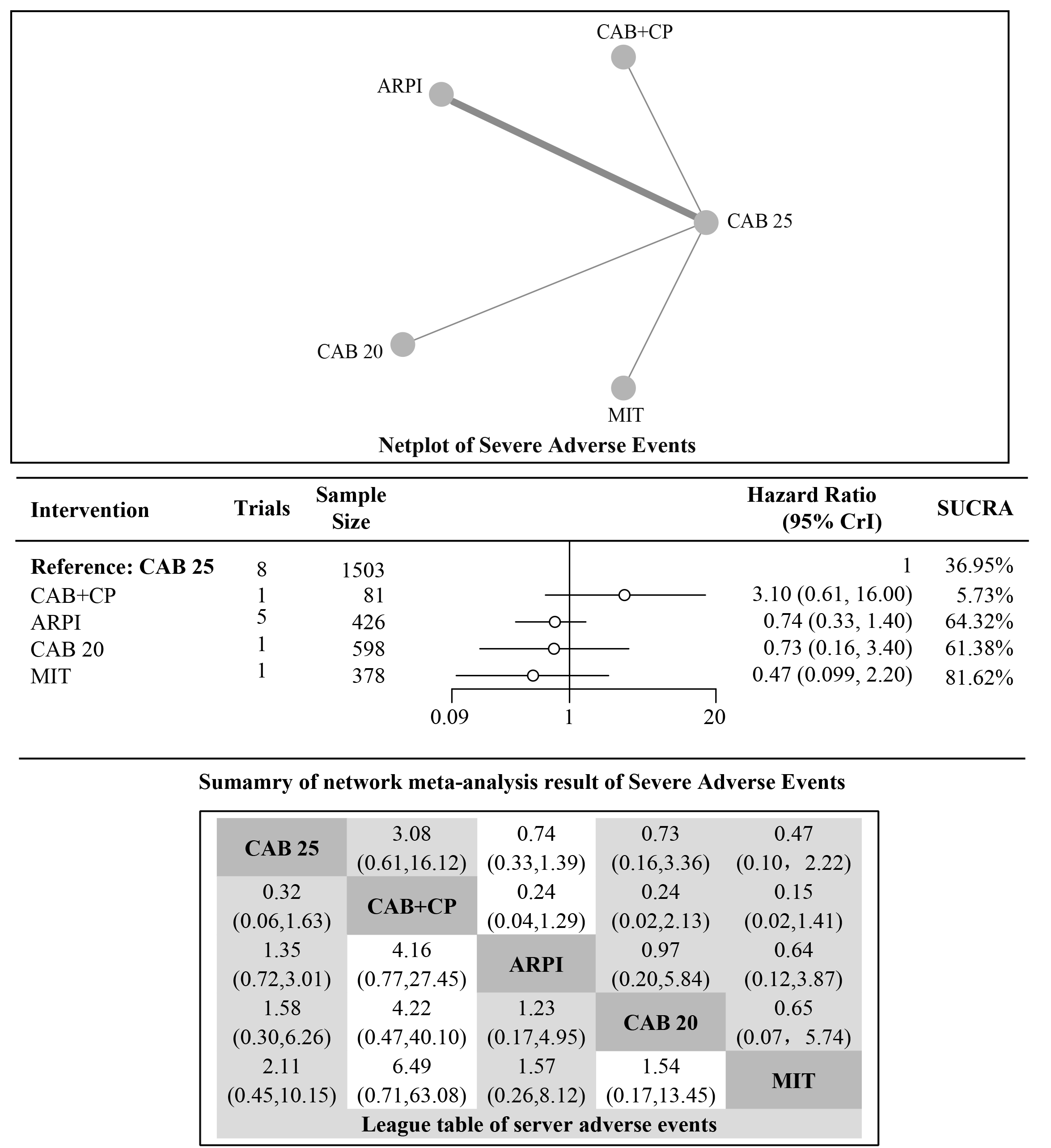

Supplement: Supplementary file 1 [file Image3.tif]

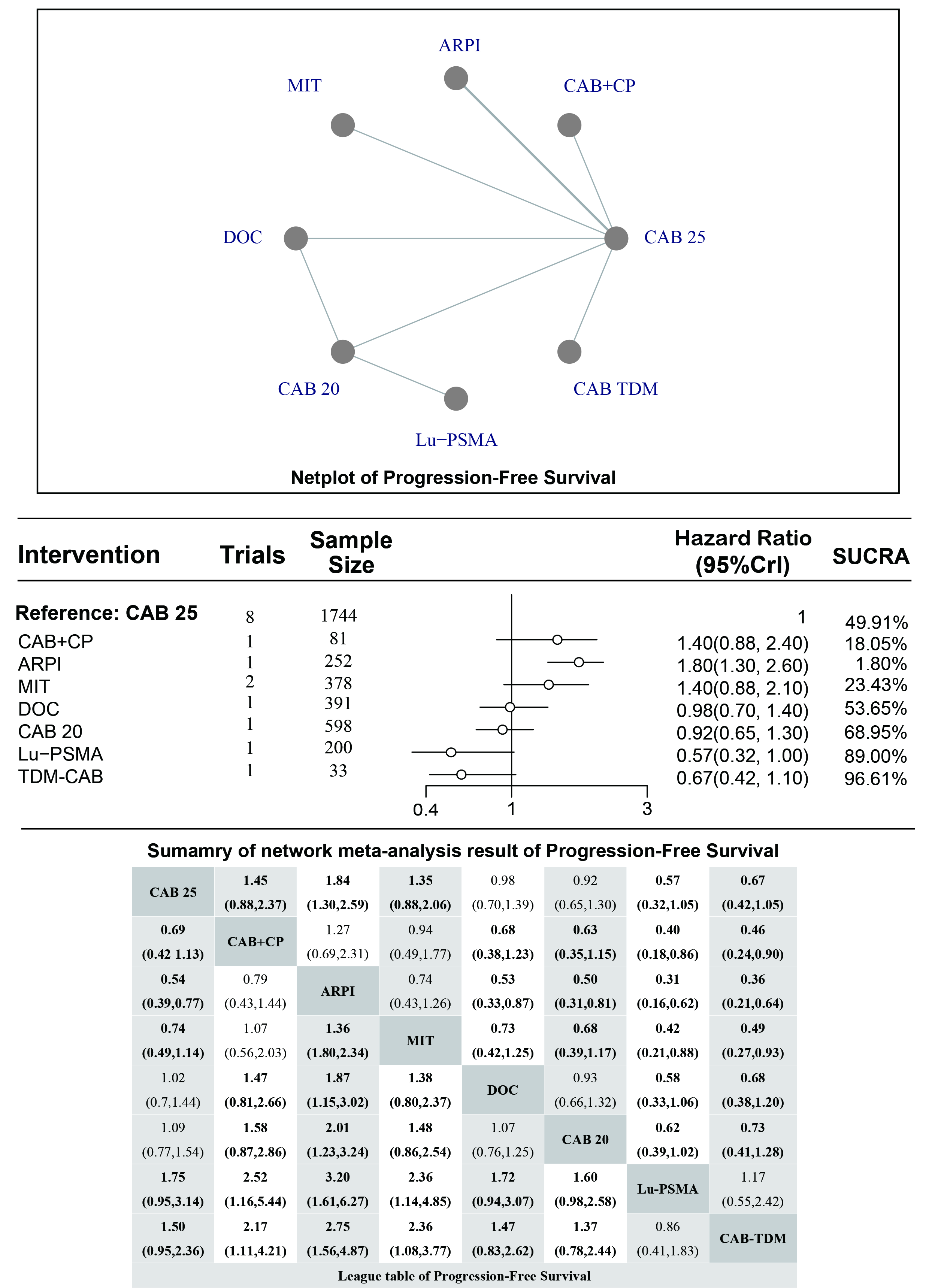

Supplement: Supplementary file 2 [file Image2.tif]

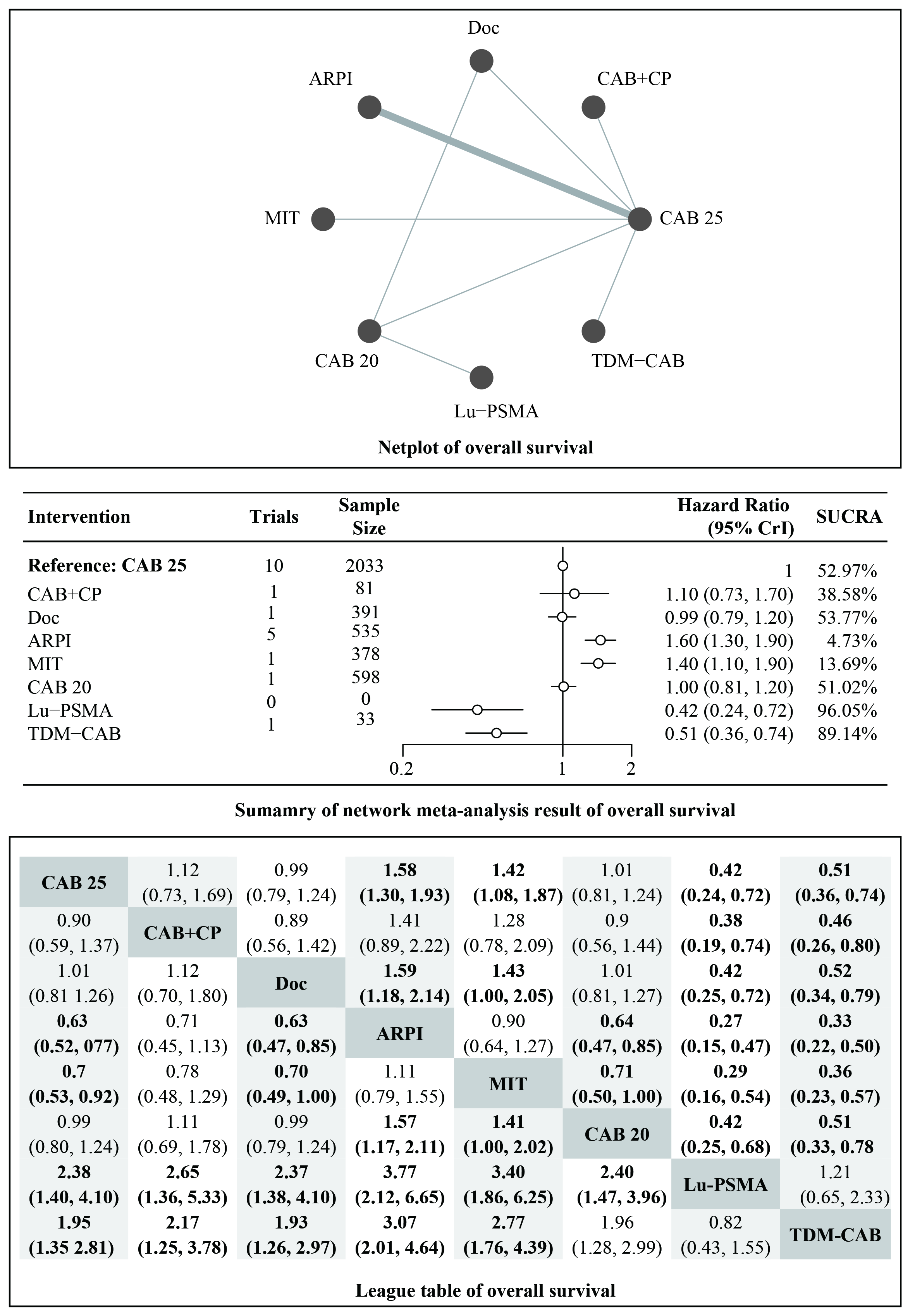

Supplement: Supplementary file 3 [file Image1.tif]
